# IOs in the BBNJ Regime Complex – the Dataset

**DOI:** 10.1016/j.dib.2023.109153

**Published:** 2023-04-17

**Authors:** Arne Langlet, Alice Vadrot

**Affiliations:** Department of Political Sciences, University of Vienna, Kolingasse 14-16, 5th Floor, 1090 Vienna, Austria

**Keywords:** BBNJ, Regime complex, Ethnographic data, International negotiations, International organizations

## Abstract

The dataset on the involvement of international organizations (IOs) in in the negotiations for a new legally binding instrument for the conservation and sustainable use of marine Biodiversity Beyond National Jurisdiction (BBNJ) under the United Nations Convention on the Law of the Sea (UNCLOS) underlies the visualizations (Figure 1,2,3) and overview (Table 1) in the publication “Not ‘undermining’ whom? Unpacking the emerging BBNJ regime complex”. The dataset describes the involvement of IOs in the negotiations through participation, making statements, being referred to by states, hosting side events and being mentioned in a draft text. Every involvement was traced to one of the package items of the BBNJ agreement, as well as to the specific provision of the draft text, where the involvement occurred*.*

**Specifications Table**

**Table:** 

Subject	Political science
Specific subject area	International negotiations
Type of data	Table
How the data were acquired	The data was obtained through participant and ethnographic observation as well as document analysis at the Intergovernmental Conferences (IGC) of the negotiations for a new legally binding instrument for the conservation and sustainable use of marine Biodiversity Beyond National Jurisdiction (BBNJ) under the United Nations Convention on the Law of the Sea (UNCLOS).
Data format	Raw
Description of data collection	The raw data was collected by a team of researchers conducting participant observation under an ethnographic approach at five IGCs of the negotiations for a new legally binding instrument for the conservation and sustainable use of marine Biodiversity Beyond National Jurisdiction under the United Nations Convention on the Law of the Sea. During plenaries, working group sessions, informals, and side events, always at least one researcher made systematic observations based on an observation matrix which predefined columns. In other words, at any time at least one researcher was following the negotiations and taking notes on his/her laptop. The observations include systematic information on the delegation of the speaker, the package item under discussion, the section and provisions of the draft text, the negotiation format, as well as a short summary and detailed the description of the statement's content. These descriptions of statements are close to a transcript of the statement. During the second and third and fifth two-weeks IGC sessions at the United Nations' New York Headquarters, we took systematic field notes. Regarding the first and fourth IGC, which the research team did not attend, we watched the proceedings via the UN webcast and took systematic field notes. In the described manner, the research team took, according to our best knowledge, notes on every statement that state or non-state actors made in the negotiations. This data was scanned for each time that an IO made a statement or was referred to by a state, and the provision in which this statement was made (and for the second case the state that made such a statement) was extracted. In this way a profile on the involvement of each IO in the negotiations was created. This information was supplemented with notes from side events, as well as all draft Treaty texts, and participant lists. This information served to add variables on the number of side events an IO hosted, the number of times it was mentioned in the draft text and the number of participants it sent to the negotiations. Further, the profile of each IO was complemented with desk research and information on inter-institutional collaboration contacts taken from the Yearbook of International Organizations (U.I.A., 2020) - a commonly used database for institutional research. Please note that the state of the data is November 2022 and thus does not include the resumed fith IGC 5.2.
Data source location	ERC Project MARIPOLDATA Department of Political Science Faculty of Social Science University of Vienna Vienna Austria
Data accessibility	[1] Repository name: dataset IOs in BBNJ regime complex; Data identification number: 11353/10.1613830; URL to repository: https://phaidra.univie.ac.at/detail/o:1613830; URL to data set: https://services.phaidra.univie.ac.at/api/object/o:1613830/download; Link to code: https://github.com/ArneLanglet/IOs-in-the-BBNJ-regime-complex
Related research article	[2] Langlet, A., & Vadrot, A. B. M. (2023). Not ‘undermining’ who? Unpacking the emerging BBNJ regime complex. *Marine Policy, 147*, 105372. https://doi.org/10.1016/j.marpol.2022.105372.

## Value of the Data

• This data is useful because it provides a comprehensive overview over the involvement of 52 different IOs in the BBNJ negotiations.
• This data can be of interest to diplomats & policy-makers; researchers; lobbyists & activists or anyone who is interested in the BBNJ negotiations and how they are linked to other IOs.
• This data can generate insights into different aspects of IO involvement in the negotiations. For example, it decsribes which IOs seem to be interested in which specific provisions of the BBNJ agreement, or which state or which alliance referred to which IO during the negotiation process.

## 1 Objective

This dataset was generated to support the visualizations in Figures 1-3 and the overview Table 1 in the publication “Not ‘undermining’ who? Unpacking the emerging BBNJ regime complex” [2]. The data adds detail to the described ways of how IOs became involved in the BBNJ regime complex and makes transparent how the graphs in Figures 1-3 in Langlet & Vadrot (2023) [2] were generated. It derives its relevance from the recognition that the BBNJ Agreement will be placed in an existing institutional landscape [3] and the observation that Article 4. on the relationship between the instrument and other IOs remained a stumbling block throughout the negotiations [4], [5] as the role of existing IOs in marine biodiversity governance remained contested. Although literature recognized and discussed the important role of regime interplay in the BBNJ case [6], such discussions and negotiations on this aspect lacked ‘additional context and detail’ [3, p. 7] as there was no overview about which IOs were involved in the BBNJ negotiations. This dataset [1] aims to fill this gap with empirics by giving detail into which IO was active, or referred to, or mentioned in which package item and provision of the draft text. It also gives contextual factors to the involvement of IOs such as which state mentioned which IO and what is the geographical scope of the mandate of the IO (among others). Following Langlet & Vadrot [2] the involvement of IOs in the BBNJ negotiations is described as a regime complex according to literature of global governance which coined the term “regime complex” to describe situations of multiple and complex regime interplay and overlap [7].

**Table 1:** Overview and explanation of variables in dataset

Variable Name	Value format	Values (detail) & source
io_abbreviation	Character	This variable contains the abbreviation of each international organization in capital letters
io_abbreviation	Character	This variable contains the full written name of each international organization in lowercase
registered_as	Character	The category under which the international organization was registered at one or more BBNJ intergovernmental conferences (https://www.un.org/bbnj/content/credentials-registration)
Geography_legal_mandate	Character	The geographical extension of the mandate of the international organization, i.e. where the international organization operates.
Type	Character	Own distinction of the international organization into the following categories: intergovernmental organization, United Nations Environmental Programme convention, United Nations agency, United Nations body
total_participants	Numeric	The aggregated amount of participants the international organization has sent to the BBNJ intergovernmental conferences 1-5 (from participant lists)
participants(1-4)	Numeric	The amount of participants the international organization has sent to each of the BBNJ intergovernmental conferences (1-4). Please note that at the time of publication the participant list for intergovernmental conference 5.2. was not yet published.
total_plenary_activity	Numeric	The aggregated amount of statements that the international organization has made in the intergovernmental conferences (1-5)
Plenary_activity_igc(1-5)	Numeric	The amount of statements that the international organization has made in any of the intergovernmental conferences (1-5)
activity_igc(1-5)_mgr	Numeric	The amount of statements that the international organization has made in any of the intergovernmental conferences (1-5) in discussions on the package item Marine Genetic Resources
activity_igc(1-5)_abmts	Numeric	The amount of statements that the international organization has made in any of the intergovernmental conferences (1-5) in discussions on the package item Area-based Management Tools / Marine Protected Areas
activity_igc(1-5)_eia	Numeric	The amount of statements that the international organization has made in any of the intergovernmental conferences (1-5) in discussions on the package item Environmental Impact Assessments
activity_igc(1-5)_cbtmt	Numeric	The amount of statements that the international organization has made in any of the intergovernmental conferences (1-5) in discussions on the package item Capacity-building and Transfer of Marine Technology
activity_igc(1-5)_crosscutting	Numeric	The amount of statements that the international organization has made in any of the intergovernmental conferences (1-5) in discussions on the package item crosscutting issues
activity_other	Numeric	The amount of statements that the international organization has made in all of the intergovernmental conferences (1-5) in discussions on no specific package item, e.g. in opening/closing statements
activity_igc(1-5)_listed_package	List of package items (comma separated numbers)	A list of the package items in which an international organization has made statements for each intergovernmental conference (1-5;9)
activity_igc(1-5)_listed_provision	List (comma separated titles of draft provisions)	A list of the provisions in the draft text during the discussion of which an international organization has made statements for each intergovernmental conference (1-5;9)
total_reference	Numeric	The amount of times any state has referred to an international organization during all the intergovernmental conferences (1-5)
reference_mgr_package	Numeric	The amount of times any state has referred to an international organization in discussion on the package item MGRs during all the intergovernmental conferences (1-5)
reference_abmt_mpa_package	Numeric	The amount of times any state has referred to an international organization in discussion on the package item ABMTs/MPAs during all the intergovernmental conferences (1-5)
reference_eia_package	Numeric	The amount of times any state has referred to an international organization in discussion on the package item EIAs during all the intergovernmental conferences (1-5)
reference_cbtmt_package	Numeric	The amount of times any state has referred to an international organization in discussion on the package item CBTMT during all the intergovernmental conferences (1-5)
reference_crosscutting_package	Numeric	The amount of times any state has referred to an international organization in discussion on the package item crosscutting issues during all the intergovernmental conferences (1-5)
reference_no_specific_package	Numeric	The amount of times any state has referred to an international organization in discussion in no specific package item (e.g. opening/closing statements) during all the intergovernmental conferences (1-5)
reference_igc(1-5)	Numeric	The amount of times any state has referred to an international organization in each of the intergovernmental conferences (1-5)
reference_igc(1-5)_listed_countries	List of country names (comma separated)	List of countries that referred to an international organization during each of the intergovernmental conferences
reference_igc(1-5)_listed_alliance	List of country or alliance names (comma separated)	List of alliance names that referred to an international organization in each of the intergovernmental conferences (1-5). The name of the alliance was used when a state spoke on behalf of an alliance, otherwise the name of the individual state was used.
reference_igc(1-5)_listed_package	List of package items (comma separated numbers)	List of package items in which any state referred to an international organization in each intergovernmental conference (1-5)
reference_igc(1-5)_listed_provision	List (comma separated titles of draft provisions)	List of the provisions in the draft text during the discussion of which any state has referred to an international organization in each intergovernmental conference (1-5)
total_draft_mentions	Numeric	Amount of times an international organization was mentioned in any of the draft texts (except of the final draft) (https://www.un.org/bbnj/content/documents)
mentions_draft(1-5)	Numeric	Amount of times an international organization was mentioned in the draft text preceding each of the intergovernmental conferences (1-5) (https://www.un.org/bbnj/content/documents)
topic_draft1_1	Character	Title of the provision in which an international organization was mentioned in the draft text preceding the first intergovernmental conference (https://documents-dds-ny.un.org/doc/UNDOC/GEN/N18/197/15/PDF/N1819715.pdf?OpenElement)
topic_draft2_(1-8)	Character	Title of the provision in which an international organization was mentioned in the draft text preceding the second intergovernmental conference (https://documents-dds-ny.un.org/doc/UNDOC/GEN/N18/413/20/PDF/N1841320.pdf?OpenElement)
topic_draft3_(1-2)	Character	Title of the provision in which an international organization was mentioned in the draft text preceding the third intergovernmental conference (https://documents-dds-ny.un.org/doc/UNDOC/GEN/N19/146/28/PDF/N1914628.pdf?OpenElement)
topic_draft4_1	Character	Title of the provision in which an international organization was mentioned in the draft text preceding the fourth intergovernmental conference (https://documents-dds-ny.un.org/doc/UNDOC/GEN/N19/372/88/PDF/N1937288.pdf?OpenElement)
topic_draft5_1	Character	Title of the provision in which an international organization was mentioned in the draft text preceding the fifth intergovernmental conference (https://documents-dds-ny.un.org/doc/UNDOC/GEN/N22/368/56/PDF/N2236856.pdf?OpenElement)
mentions_draft5.1	Numeric	Amount of times an international organization was mentioned in the draft text that was circulated at the end of week 1 at intergovernmental conference 5
topic_draft5.1_1	Character	Title of the provision in which an international organization was mentioned in the draft text at the end of week 1 at the fifth intergovernmental conference
amount_side_events_hosted	Numeric	The amount of side events that an international organization has hosted at all the intergovernmental conferences (1-3)
topical_focus(2-4)	Character	A categorization of the topical focus on which an international organization works as indicated in their mission statement/mandate/establishing treaty
grouped_topical_focus	Character	An overarching categorization of the topical focus on which an international organization works as indicated in their mission statement/mandate/establishing treaty
source_topical_focus	Character	The source for the previously made categorization of the topical focus
link_topical_focus	Weblink	A weblink to the source of the topical focus
year_entry_into_force	Date (YYYY)	The year in which the legal document establishing the international organization entered into force
year_open_for_signature	Date (YYYY)	The year in which the legal document establishing the international organization opened for signature
total_members	Numeric	The amount of member states an international organization has (ratified)
member_states	List of country names	A list of the member states of each international organization
organizational_contacts_(uia)	List of organizational contacts to other international organizations	The information the Yearbook of International Organizations gives under the section “Relations with Inter-Governmental Organizations” which is based on an annual survey with international organizations (https://uia.org/yearbook)
staff_amount	Numeric	Amount of staff that works for an international organization (where indicated on its website)
budget_(in_mio_usd)	Numeric	The yearly budget of an international organization (where indicated on its website)
office_location	Character (country names)	The country in which the headquarter of the international organization is located.

## 2 Data Description

The dataset contains the full profile of all IOs (as defined in Langlet & Vadrot, 2023) involved in the BBNJ negotiations. As such it complements Table 1 in the publication. This section describes in detail each of the variables, what type of values it contains and their sources/ how they were generated.

The intergovernmental conferences (IGC) of the BBNJ negotiations are numbered from 1-5.

The package items are numbered as follows: 1 = Marine genetic resources (MGRs); 2 = Area-based management tools including marine protected areas (ABMTs/MPAs); 3 = Environmental impact assessments (EIAs); 4 = Capacity building and the transfer of marine technology (CBTMT); 5 = Crosscutting issues; and 9 = general. Please note that the cut-off date of the data is November 2022 and thus it does not include the resumed fith IGC 5.2.

## 3 Experimental Design, Materials and Methods

The data was acquired through the following steps: 1. We took systematic field notes through collaborative ethnographic fieldwork during the second, third and fifth two-week IGC sessions at the United Nations' New York Headquarters. Regarding the first and the fourth IGC, which the research team did not attend in-person, we took systematic fieldnotes using the same methodology based on online participation and digital ethnography. This data was supplemented with notes from side events, as well as all draft texts, and participant lists.

2. A list of IOs was created based on the definition of an IO as an organization that contains a) a normative framework (normally codified in a treaty), b) member states (or other IOs as constituting members), and c) a body such as a secretariat with staff, budget, and a registered office embodying the normative framework.

3. Using R programming language, the bulk of collected ethnographic field notes, draft texts and participant lists was then scanned for any reference to any of the IOs included in the list. All IOs are in the dataset because 1) states refer to the IO in relation to provisions of the draft text; 2) IOs themselves made statements or send representatives in the negotiations, and 3) IOs were mentioned in a version of the draft text. For each reference, statement, or draft mention, the relevant provision was traced to determine where exactly these IOs came into contact with the BBNJ process.

4. Using this data, we created a profile of each IO involved in the BBNJ process. This profile was complemented with desk research to add general information on the IO at hand.

The code written in R programming language that was used to process and analyze the ethnographic fieldnotes to produce the described dataset can be found under: https://github.com/ArneLanglet/IOs-in-the-BBNJ-regime-complex.

## Ethics Statements

The dataset was generated as part of the ERC Research Project MARIPOLDATA led by Assoc. Prof. Alice Vadrot. The research methodology and data collection methods were reviewed and approved by the Ethics Committee of the European Research Council Executive Agency, Unit B1 – Ethics Review and Expert Management on 05/10/2018. In addition, the research was reviewed and approved by the Ethic Commission of the University of Vienna on 20/10/2018.

The data in this dataset is fully anonymized and does not contain any personal or human data. Before the ethnographic fieldwork was undertaken, an email was sent to the secretariat organizing the BBNJ conferences, informing them about the project, its objectives and the data to be collected. An information sheet summarizing the project and an informal consent form was attached to the email. The secretariat was asked to inform participants about the study/information. Therefore, material (leaflets) was available at an information desk.

## CRediT authorship contribution statement

**Arne Langlet:** Conceptualization, Methodology, Software, Data curation, Writing – original draft, Visualization. **Alice Vadrot:** Conceptualization, Methodology, Supervision, Writing – review & editing.

## Declaration of Competing Interest

The authors declare that they have no known competing financial interests or personal relationships that could have appeared to influence the work reported in this paper.

## Data Availability

dataset IOs in BBNJ regime complex (Original data) (https://phaidra.univie.ac.at/detail/o:1613830).
